# Supplementary material for: Are carbon emissions trading and green financial instruments synergistic? -Comprehensive quantitative research based on content analysis
Source: PLoS One. 2024 Mar 7;19(3):e0298601. doi: 10.1371/journal.pone.0298601 (PMC10919601; doi:10.1371/journal.pone.0298601)
Supplement: S1 Data — (ZIP) [file pone.0298601.s001.zip › Data/地级市Z_F工作报告.zip!/╣π╢1⁄2/╒╪╟∞╩╨/└┤╫╘╣1⁄2╓┌║┼í╛┬φ┐╦╩2╛▌═°í┐_2004.docx]

各位代表：

　　我代表市人民政府向大会作政府工作报告，请予审议，并请市政协各位委员和其他列席人员提出意见。

去年工作回顾

　　２００３年，在中共广东省委、省人民政府和中共肇庆市委的正确领导下，在市人民代表大会及其常委会和市政协的监督支持下，市政府按照市第九次党代会的战略部署，统一思想，积极进取，求真务实，锐意创新，一手抓抗击"非典"，一手抓经济建设，迅速掀起新一轮发展热潮，推动国民经济保持良好的增长态势，社会各项事业取得新的进步，实现了 "一年打基础"的工作目标。

　　——积极实施东进战略，国民经济发展势头良好

　　经济综合实力增强。三大经济板块建设全面推进，中心板块新经济产业逐步上规模、上水平，东南部加工制造业经济板块成为承接珠三角产业扩张转移的先发地区、吸引制造业集聚发展的新兴基地，山区县域经济板块工业化、城镇化、农业产业化步伐加快；外资经济、民营经济、园区经济不断发展壮大；体制、科教、城市化三大平台建设取得新成效，有力地推动了国民经济快速健康发展。肇庆市生产总值完成５０１．１５亿元，比上年增长１１．２％，其中第一产业增加值增长５％，第二产业增加值增长１６．５％，第三产业增加值增长１０．６％。产业结构得到优化，三次产业比例从上年的２８．１∶３６．２∶３５．７调整到２６．５∶３８．８∶３４．７。人民生活水平不断提高，城镇居民人均可支配收入８０４０元，农村居民人均纯收入４１５０元，扣除物价因素分别比上年实际增长１０．７％和３．２％。各项经济社会发展指标均达到或超过市十届人大一次会议确定的目标。

　　工业经济发展提速。工业强市战略进一步落实，新型工业化稳步推进，工业结构进一步优化，质量效益不断提高。全市新登记各类工业企业１６８１户，新增工业注册资本３０多亿元。高新技术产业和传统优势产业得到发展壮大，风华集团、星湖科技公司等重点骨干企业增长势头良好。园区经济进一步壮大，高新区大旺工业园等重点园区成为新的经济增长点。企业技术改造得到加强，全年技改竣工项目２６项，总投资５．０２亿元。全市工业增加值比上年增长１７．３％，规模以上工业销售产值增长１５．９％，实现税利增长９．４％，工业经济效益综合指数提高１３．３个百分点。全市供电量比上年增长１８．２％。

　　农村经济稳定发展。农村工业化、城镇化和农业产业化步伐加快，县域经济发展道路拓宽。以扩大招商引资和加强园区建设为手段，促使山区资源型产业不断发展壮大，形成了封开、德庆的打火机生产，德庆、怀集的林产化工和生态旅游，广宁鼎丰纸业等山区特色产业。中心镇规划建设工作全面铺开，产业集聚和吸纳农村富余劳动力的能力增强。财政对农村经济的支持力度加大，共投入２．９６亿元支持农业产业化、农业科技推广和水利设施建设。调整优化农业结构，大力发展特色农业，积极抓好肉桂、柑桔、肉鸡、食用笋竹等特色农业基地建设，十大农业主导产业和十大名牌产品规模不断扩大。各类产业化组织、专业批发市场和年产值５００万元以上的农业龙头企业分别发展到１５４个、１３个和７３户，"三高"农业基地面积达７４０万亩。创办了６个省级农业标准示范区。林业生态建设进展顺利，超额完成绿化造林任务，启动了北岭山林分改造二期工程，筹建省、市级自然保护区８个。城乡水利减灾防灾工程建设深入推进，景丰联围等重点江堤、各县（市）区县城防洪工程和中小型水库除险加固达标建设进展顺利。全市农业总产值比上年增长５．７％。

　　民营经济发展势头良好。认真落实省加快发展民营经济的工作部署，制定实施了我市关于加快民营经济发展的政策和措施。加强了对民营经济的引导，提高了政府服务水平，民营经济发展环境进一步优化。千方百计激活本地民资和扩大利用外地民资，扶持发展民营科技企业和民营科技工业园区，民营经济实力壮大，质量提高。年末全市个体工商户、私营企业达到７．２２万户，比上年末增加１０８３户，其中私营企业登记注册户数和注册资金分别比上年末增长２６．２％和５３．４％。个体私营企业入库税收总额首次突破４亿元，比上年增长１２．２％；民营经济增加值比上年增长１６．４％，占肇庆市生产总值的３３．９％。

　　旅游业等第三产业实现恢复性发展。大力扶持受"非典"影响较大的旅游、交通运输、餐饮等第三产业。以构建千里旅游走廊为主线，加大景区景点的整合和开发力度，加强旅游基础设施建设，组织系列宣传推介活动，使旅游业在"非典"过后迅速恢复。全年城市接待过夜旅游者人数和旅游收入分别比上年下降８．６％、８．２％。消费环境进一步改善，消费结构逐步升级，消费需求平稳上升，城市居民家庭人均消费性支出增长１１．４％，形成了汽车、住房等新的消费热点。电信服务不断推出新业务，商贸、餐饮和其他服务业有新的发展。房地产开发势头良好，完成开发投资比上年增长１２．８％，商品房销售面积和销售额分别增长２９．８％和４４．２％。全市社会消费品零售总额１７１．１９亿元，比上年增长１０．７％。

　　财税、金融平稳增长。税收征管得到强化，财政收入稳步增长。全市地方财政总收入３２．５２亿元，总支出３２．３２亿元，实现收支平衡。其中地方财政一般预算收入完成１４．４４亿元，比上年增长１３％；地方财政一般预算支出３１．８９亿元，增长１８．５％。金融部门增强了对地方经济发展的支持，各项存款余额和贷款余额分别比上年末增长１６．２％和６．８％。全市城乡居民储蓄存款余额２５６．７２亿元，增长１４．１％。

　　——招商引资取得历史性突破，基础设施建设步伐明显加快

　　招商引资掀起新热潮。不断扩大招商引资地域，创新了招商引资方式，搭建起国际化、市场化的招商平台，做到扩大利用港澳台、美、日资本和拓展利用欧洲、韩国等资本并举，利用境内和境外资本并举，吸收外资水平明显提高。成功举办重点项目签约仪式、"肇庆金秋"经贸洽谈会和承办２００３年广东省华侨农场招商洽谈会，其中"肇庆金秋"经贸洽谈会签约和奠基项目投资总额折合人民币５２６亿元。引进了亚洲铝业、正宝山铜业等一批实力雄厚、关联带动能力强的大企业、大项目。全市新批注册合同外资额比上年增长１８５．９％，实际利用外资６．８２亿美元，增长２５％。扎实推进全国电子口岸试点工程建设，通关效率进一步提高，外贸出口再创历史新高。全年外贸出口总额１１亿美元，比上年增长２２％，其中一般贸易出口增长３９．２％。

　　基础设施建设速度加快。城乡规划编制不断完善，完成了中心城区主要路网规划调整论证。中心城区东扩南连建设开始启动，城东新区系列项目奠基。加大土地储备力度，推动融资方式多元化，扩大利用了各类资金。城区道路建设和路灯改造工程逐步铺开。宋城西路开通工作进展顺利，跃龙涌覆盖工程基本完成。肇庆新港一期工程、西江大桥扩建工程和景丰联围除险加固工程正在抓紧进行。三水至怀集高速公路已规划立项报批。全面完成了省道砂土路改造任务。县省道上等级改造工程和乡镇通行政村公路硬底化工程进度加快。固定资产投资快速回升，全社会固定资产投资完成１１４．７５亿元，比上年增长１７．６％，其中基本建设投资增长１７．９％；全市新开工项目计划投资总额１２３．５亿元，增长２１８．６％。

　　环境综合整治力度加大。创建国家环境保护模范城市活动深入开展。编制了《肇庆市治污保洁工程实施方案》，以西江水环境为重点，开展环境综合整治，全市一级饮用水源保护区基本实现污染物零排放，主要江河湖库地面水和饮用水水源达到水环境功能区的标准。城区空气质量良好。城区交通干线噪声和区域环境噪声平均值达到标准。市污水处理厂二期工程进展顺利，高要、四会等县（市）区污水处理厂筹建工作已经启动。

　　——体制改革不断深化，社会保障和就业形势进一步好转

　　国有企业改革取得新突破。以市直国有企业为重点，加大了企业产权改革的力度，多渠道筹集改革成本，积极引入各类资金参与企业改革，生产性企业的存量资产得到有效盘活。方大气动、动力配件、香料厂等企业已基本完成产权改革，化纤厂、物资集团、南华集团、羚羊集团等困难亏损企业依法实施了关闭破产。市直实施产权改革的企业共４５户，合计筹措改革资金１．９２亿元，安置职工１３１４９人，盘活资产５．６２亿元。

　　政府行政管理体制改革不断深化。完成了第二轮政府行政审批制度改革，审批项目由６５０项减少到２２６项。公共财政体制改革进一步深化。开展了公共资源及政府特许经营项目市场化运作试点，实行国有土地和河砂等矿产资源公开招投标，收到良好效果。事业单位人员聘用制度改革稳步推进。

　　社会保障和就业工作得到加强。社会保险覆盖面继续扩大，实现了社会保险全市统筹，到去年底止，全市享受社会保险待遇１０．２９万人，社会保险待遇支付率１００％。建立就业和再就业目标责任制，省、市、县三级劳动力市场实现信息联网，扶持再就业政策得到落实，在全省率先建立下岗失业人员小额贷款担保中心。全市各级财政已安排再就业专项资金１５１８万元，新增城镇就业岗位２．７万个，年末城镇登记失业率２．９％。初步建立劳务输出培训机制，组织输出农村富余劳动力８．１３万人。

　　——"科教兴市"战略进一步落实，社会各项事业取得新成绩

　　科技工作成效明显。全社会科技意识不断提高，科技对经济发展的推动作用进一步增强。全市投入科技领域的财政资金９２７２万元。组织申报和实施了８０项省级以上各类科技计划。被认定为省市高新技术企业１２家，省级民营科技企业１３家。有３项技术获得国外专利。组建省、市级工程中心６家，省级以上工程技术中心数量居于全省地级市前列。开展了省、市级专业镇技术创新试点和市级民营科技园创建工作，高要金利镇和端州黄岗镇被列入省级专业镇创新试点。科技合作领域逐步扩大。中国与巴西软件合作开发项目落户肇庆。"数字肇庆"建设开始实施。开通了疾病疫情信息网络系统。

　　教育事业蓬勃发展。高度重视发展教育，全市共筹措７．２亿元资金投入教育。以县为主的基础教育管理体制和中小学教育收费"一费制"进一步完善，普及九年义务教育不断巩固提高。中小学布局调整加快，累计调整学校５７９所，扩大校舍建筑面积约３０万平方米。教育信息化上新水平。普通高中办学规模进一步扩大，高考上线人数再创历史新高。高等教育、中等职业教育、农村成人教育、学前教育和特殊教育有新的发展。社会力量办学在不断规范中发展壮大。

　　文化、体育发展水平提高。着手开展文化名市建设研究。文化基础设施建设力度加大。文物普查、保护工作得到加强，宋城墙西段进行了保护开发，白沙龙母庙主体修葺复原工程竣工。文艺创作演出繁荣，群众文化活跃，社会科学、新闻出版、广播电视等文化事业得到加强，广播人口覆盖率和电视人口覆盖率分别达到９８％和９６．６％。建成了肇庆城区全民健身广场，成功举办市第七届运动会，竞技体育水平得到较大提高，我市运动员获得２项世界冠军、７项亚洲冠军和３６枚全国重大比赛金牌。

　　公共卫生建设不断加强。高度重视和积极应对"非典"疫情，认真落实各项防控措施，投入"非典"防治资金１１００万元，全力以赴救治病人，取得了抗击"非典"斗争阶段性胜利，稳定了经济社会大局。建立突发公共卫生事件应急处理机构和防治体系，加强公共卫生监督管理，组建了市卫生监督所和市疾病预防控制中心。成功主办国际中医药学论坛。职业卫生管理制度得到落实。全市乡镇基本建立农村合作医疗保障制度，有１１７．６９万人参加农村合作医疗，完成了省下达的目标任务。重视农村落后地区卫生环境建设，扶持了一批村庄改水改厕。

　　其他社会事业稳步推进。完成了人口与计划生育工作任务，人口自然增长率６．１７‰，比上年下降０．４７个千分点。城乡居民最低生活保障制度更加健全，基本实现动态管理下的应保尽保。离休干部离休费和医药费保障机制进一步完善。救灾和扶贫工作扎实有效开展。社会救助管理全面启动。社区建设步伐加快。国防动员、兵员征集、民兵预备役和双拥优抚安置工作进一步加强。殡葬改革得到落实。海外联谊和港澳台联谊工作进一步拓展，建立友好合作关系的国外城市不断增多。充分发挥侨务资源，争取各类捐款２７８４万元。安全生产形势稳定。审计、统计、妇女儿童、民政、民族、宗教、地方志、质量监督、气象、防震、人防、档案、残联等工作取得新成绩。

　　——民主法制得到加强，精神文明建设取得新发展

　　民主法制建设继续推进。自觉接受民主监督，广泛听取各方面对政府工作的意见。坚持向人民代表大会及其常委会报告工作、向人民政协通报情况，已办结市人大会议议案１件和省、市人大代表建议、批评、意见９９件，已经解决或列入计划逐步解决的７８件，办理市政协提案１０３件。加强了与工会、共青团和妇联等群众团体的联系。重大事项社会公示和听证制度逐步落实，科学化、民主化决策机制进一步完善。全民法制宣传教育深入开展。法律服务工作得到加强。依法行政水平进一步提高，行政执法监督和行政复议工作更加规范。村民自治、社区居民自治和企事业单位民主管理制度不断完善。

　　社会治安综合治理不断加强。实施了社区警务规范化建设，进一步完善"四警联动"的快速反应治安防控体系和治安联防网络。坚持不懈防范和打击"法轮功"等邪教组织。开展了打击恶性暴力犯罪和"两抢"、"两盗"、"六合彩"赌博、非法传销等一系列专项行动，实施了"禁毒０３工程"，治安状况持续好转。深入开展人民调解工作，积极化解和妥善处理人民内部矛盾特别是涉及群众切身利益的矛盾。

　　精神文明建设有新气象。深入贯彻中共十六大和十六届三中全会精神，掀起了"三个代表"重要思想学习教育活动的新高潮。大力宣传抗击"非典"先进事迹。组织开展了"现代肇庆人精神"大讨论。评议并确认了肇庆新八景。认真组织实施《公民道德建设纲要》。文明社区、文明村镇、文明行业和文明单位创建活动深入开展，科教、文体、法律、卫生"四进社区"工作取得新成效。全市安全文明小区覆盖率达８８．４％。

　　——政府服务不断改进，"十项民心工程"逐步落实

　　政府机关作风建设不断加强。各级政府和领导干部在抗击"非典"斗争中经受住严峻考验，应对"突发"事件能力增强。按照"为民、务实、廉洁"的要求，扎实开展政府机关作风建设年活动，公务员队伍服务意识、办事效率得到提高。政务公开、电子政务建设取得新进展，行政服务水平不断提高。认真落实领导干部岗位目标责任制和廉政建设责任制，加强了从源头上防治腐败的制度建设，严肃查处了一批违法违纪案件。重视群众来信来访，认真纠正了损害群众利益的不正之风。

　　"十项民心工程"开始实施。认真落实农民增收减负工程，"三并一减"（并镇、并村、并校、精简人员）稳步实施，农村税费改革取得阶段性成果，市财政支出税费改革配套资金１４７０万元，年减轻农民负担１．７４亿元，农民人均减负率８０％以上。实现了县（市）区为单位的城乡用电同网同价。教育扶贫工程扎实推进，建立了助学贷款风险准备金。保护外来工合法权益工作得到有效加强。全民安康工程加快实施。城镇医疗保险工作完成了预定的目标任务。逐步解决农村缺水地区饮水难问题。加快建设城乡水利防灾减灾工程，对台风、洪水、地震等自然灾害的防灾救灾监测网和预防预警机制进一步健全。

　　回顾总结去年工作，我们清醒地认识到：我市经济增长速度仍低于全省平均水平，质量和效益不高；工业化和城市化水平比较低；区域发展不平衡，加快发展山区县域经济的任务艰巨；一些基础设施建设工程未能达到计划进度要求，部分已签约项目尚未有效落实；社会保障体系建设有待加强，农民收入增长缓慢，城镇居民总体收入水平不高，部分下岗职工生活比较困难；国企改革的任务仍很艰巨，就业和再就业压力大；教育、卫生等社会事业发展水平仍较低。特别是政府工作的责任机制尚未完善，检查督促力度不够；一些部门大局意识淡薄，相互之间沟通协调不够；少数领导干部缺乏进取精神和务实作风，一些公务员尤其是中层干部缺乏工作的责任感和紧迫感，服务意识不强，创新精神不足。这些问题我们将在今后的工作中努力加以改进。

　　各位代表，在国际局势风云变幻、国内经济竞争激烈、"非典"突如其来造成严重影响的情况下，我市经济社会保持良好的发展态势，为实现跨越发展打下了坚实的基础。我们所取得的成绩，是努力实践"三个代表"重要思想，深入贯彻党的十六大、十六届三中全会精神，按照中央和省的工作部署开拓创新的结果；是市委审时度势，抢抓机遇，确立新的发展思路，实施新的发展举措的结果；是全市人民共谋发展、奋力拼搏、真抓实干的结果；是各级人民代表大会及其常委会和人民政协监督支持的结果；也是驻肇单位、武警官兵、港澳台同胞、海外侨胞和社会各界关心支持的结果。在此，我代表市人民政府，向全市工人、农民、知识分子、干部职工、外来务工人员、驻肇单位、驻肇部队、武警官兵以及各界人士，致以崇高的敬意！向关心和支持我市建设的外来投资者、港澳台同胞、海外侨胞和国际友人表示衷心的感谢！

今年工作安排

　　今年是全面贯彻中央和省各项决策部署，实现市第九次党代会确定的"三年见成效，五年大发展"目标的关键一年。当前，国际产业结构调整加快，国际资本流动加速，我国认真履行加入世贸组织的承诺，经济增长势头良好，增强了国际经济合作和外商投资的信心，国外大财团、大企业积极寻求在中国投资发展的机会。内地与港澳建立更紧密经贸关系，有利于我市大力发展外源型经济；省委、省政府实施发展珠三角、推动大珠三角、谋划泛珠三角战略部署，有利于我市承接发达地区的产业扩张和产业转移，进一步融入珠三角特别是广佛经济圈的发展潮；全市上下已经形成团结奋进、开拓创新的发展氛围，为做好新一年工作打下坚实的基础。但是，我国加入世贸组织的过渡期到２００５年就要结束，政府和企业运作模式将面临很大的挑战；珠三角核心区通过资源整合极大地提高整体竞争力，周边地区借助发达地区经济辐射，形成你追我赶、百舸争流的竞争局面。面对机遇和挑战，我们要坚定信心，振奋精神，求真务实，开拓创新，进一步增强加快发展的紧迫感和使命感，努力开创工作新局面。

　　今年政府工作的指导思想是：以邓小平理论、"三个代表"重要思想为指导，认真贯彻党的十六大和十六届三中全会精神，按照市第九次党代会和市委九届二次全会的各项工作部署，狠抓落实、推进改革、注重效益。树立和落实科学的发展观，不断完善社会主义市场经济体制；积极实施东引西连战略，着力抓好招商引资和交通、能源基础设施建设，全力打造三大经济板块；优先发展工业，切实解决"三农"问题，做大做强旅游业，促进三大产业协调发展；坚持以人为本，努力改善人民群众特别是农民、城镇困难职工的生活；继续优化生态环境，高度重视文化名市和教育强市建设，加快社会各项事业发展；积极推进民主法制建设和精神文明建设，努力提高行政服务水平，为全面建设小康社会和率先基本实现现代化打下坚实的基础。

　　２００４年全市主要经济预期目标安排如下：肇庆市生产总值增长１３％，一、二、三产业增加值分别增长５．５％、１７．７％和１３．５％，全社会固定资产投资增长２０％，社会消费品零售总额增长１３％，地方财政一般预算收入增长１３．５％，外贸出口总额增长８％，实际利用外资按新口径增长３５％（按旧口径增长２２％），城镇登记失业率控制在３％左右，城镇居民人均可支配收入增长１０％，农村居民人均纯收入增长４．５％，人口自然增长率控制在８．３‰以内。实现今年经济社会发展预期目标，必须坚持改革与发展并重，做到速度和结构、质量、效益协调发展；必须狠抓重点，坚定不移地把工业放在牵动全局地位，突出抓好招商引资、民营经济和交通能源、城市基础设施建设；必须重视区域协调发展，关注扶持落后地区和困难群众；必须坚持执政为民，进一步转变工作作风，着力解决好当前经济社会发展中的突出问题和薄弱环节；必须坚持"三个文明"一起抓，推动经济社会实现可持续发展。重点做好以下工作：

　　一、狠抓工作落实，加快经济跨越发展

　　（一）落实工业强市战略，推动产业结构优化升级

　　做大做强支柱产业。做好产业发展规划研究，选准产业发展方向，迅速形成规模，壮大经济总量，提高质量和效益。主动承接广佛经济圈产业扩张转移，努力建设成为珠三角产业配套基地。大力发展电子信息、生物制药、软件工程为重点的高新技术产业，积极引进国内外信息技术、人才和项目，加快建设软件产业出口基地和中国与巴西两国科技部合作软件开发项目。扶持电子设备、金属制造等规模产业，发展壮大以风华集团为核心的电子设备制造业和以亚洲铝业为重点的金属制造业。大力整合汽车零配件产业，以动力配件、本田金属、鸿图科技、四会连杆、怀集汽配等骨干企业为龙头，推动汽配产业加快发展。改造提升造纸、林化、饮料、建材等传统优势产业。增强名牌效应，扶持发展一批带动能力强、拥有自主知识产权的重点行业、重点企业和重点产品。支持风华、星湖、蓝带等龙头企业通过完善现代企业制度，整合内部资源，加强对外合作，增强核心竞争力。引导和扶持各类企业加大技改投入，强化内部管理，提高经营效益。加快实施信息化工程，发展电子商务，鼓励企业扩大利用信息技术创新经营管理模式、技术研发模式和市场营销模式。

　　加快工业园区开发建设。加强园区统筹规划、资源整合和功能配套，切实抓好大旺工业园、金渡工业园、南江工业园等重点园区的开发和招商引资，支持亚洲铝业城、金陶工业城和加工贸易集中监管园区等重点项目建设。积极引入多元化投资开发主体，鼓励社会投资者参与园区开发。规范完善园区开发建设和招商引资政策，切实降低营商成本。抓好园区基础设施和配套设施建设，创造优良的投资环境。加强园区产业规划、引导，发展一批规模大、高成长和产业链延伸能力强的企业，提高园区产业集聚度和综合竞争能力。

　　积极推进农村"三化"和县域经济发展。认真贯彻落实中共中央２００４年１号文精神，把"三农"问题作为当前工作的重中之重，积极推进农村工业化、城镇化和农业产业化，努力形成以工业化为核心、"三化"互动互促的县域经济发展新路子。落实各类扶持政策，切实帮助农民增收减负。加强农村劳动力的技术培训，促进农村富余劳动力转移就业，发展劳务经济，支持农民自主创业增收。加大对农村发展的投入，建立健全财政支农资金的稳定增长机制，鼓励和引导各类社会资本投向农业和农村。加大农业结构调整力度，稳定和提高粮食生产，发展名优高效产品，扩大十大农业名牌产品规模。推动农业标准化生产，提升特色农产品的质量和档次。积极实施"绿色证书工程"，提高农业科技普及率。构建农业服务体系，大力扶持发展农产品加工龙头企业，发展农产品流通市场和销售渠道，促进农产品增值。在稳定完善农村基本体制和经营制度的基础上，鼓励引导农民依法、自愿、有偿流转土地承包经营权，积极稳妥推动集体土地使用权流转。完善土地征用程序和补偿机制，保障农民合法权益。加强农村基础设施建设，掀起农田水利基本建设新高潮。严格实施中心镇规划，加快中心镇特别是示范中心镇建设。建立完善专业镇技术创新服务体系，培育发展专业镇和特色产业。帮助山区增强承接珠三角产业转移能力，发展资源型优势产业、优势企业和优势产品。

　　大力发展以旅游业为重点的第三产业。按照"景点企业化、管理行业化、资源市场化、产权多元化"的原则，加快推进旅游管理体制改革，有效整合全市旅游资源，变资源优势为产业优势。加强城市品牌形象推介，突出休闲度假旅游主题，不断丰富旅游的文化内涵。加大资金筹措力度，打造星湖风景名胜区、盘龙峡景区两个精品景区，新建鼎湖山景区专用停车场，以科学的态度开发建设羚羊峡和砚洲岛，改造完善高要广新农业生态园、四会贞山、广宁竹海大观、怀集燕岩和世外桃园、封开天下第一石和白石岩等景区景点，构筑区域性的肇庆千里旅游走廊热线。发展现代物流业、服务业、房地产业。推进城市商业网点规划和建设，扶持发展一批大型连锁经营企业、物流企业和中高级批发市场。发展社区服务业。推动城区房地产业向东拓展，筹建国际会议中心、专业会展中心和五星级宾馆。改善消费环境，维护消费者合法权益。培育和扩大住房、汽车、通讯、教育、健身、文化等消费热点，推动消费结构升级，增大消费总量。积极适应国家调整出口退税政策，引导企业优化出口产品结构和市场结构，扩大本地高新技术产品和传统优势产品出口，拓展新兴出口市场，培育发展骨干外贸企业和民营外贸企业，推进电子化通关建设，促进外贸出口稳定增长。

　　（二）落实东引西连战略，推动经济协调发展

　　再掀招商引资热潮。乘势而上，全力以赴，继续扩大招商引资，抓好已签项目的落实。继续抓好对日、韩、美、台招商，扩大对欧盟特别是法国、爱尔兰和巴西等国的招商，着力引进一批在国内外有影响力的大企业、大集团。抢抓内地与香港、澳门实施更紧密经贸关系安排的先机，积极引入港澳优势产业。高水平承办好２００４＇粤台经济技术贸易交流会，精心组织"肇庆金秋"经贸洽谈会，力争再引入５００亿元的外来投资。对去年签订和今年新签的招商引资项目，切实抓好跟踪落实。建立招商引资任务和项目跟踪落实工作责任制，加强督查，严格考核，确保落实，使已签订的意向尽快转为投资合同，已签订的投资合同尽快注资到位，已注资的项目尽快动工投产，切实提高招商成效。

　　大力发展民营经济。继续落实加快民营经济发展的政策措施，加强政府服务，放宽市场准入条件，努力降低营商成本，为民营企业发展创造良好的法制、政策、政务、市场和舆论环境。抓好民营经济服务体系建设，推动建立行业组织和技术创新中心，鼓励和支持民营企业制度、技术、管理创新，拓展市场，发展壮大。积极帮助民营企业解决贷款难问题。大力扶持科技型、外向型、吸纳下岗人员再就业型、农产品加工型民营企业发展。

　　加快以立体交通体系为重点的基础设施建设。主动参与"泛珠三角"区域经济合作，充分发挥我市处于"东引西连""龙颈"部位的区位优势，大力推进与珠三角核心地区和大西南地区基础设施一体化，构筑我市陆路、水路和航空立体交通体系。把建设通广州新机场快速干线放在首要位置，力争年内动工；做好珠三角外环高速公路肇庆段前期准备工作，尽快打通"往东赶"的出口，缩短肇庆与珠三角核心区的时空距离，实现资源共享，使广州新机场也成为肇庆的机场，鼎湖山也成为珠三角的鼎湖山。积极推进广贺高速公路三水至怀集段建设，主动配合做好太澳线（太原--澳门）和昆汕线（昆明--汕头）两条高速公路肇庆段的前期筹备工作，着手进行３２１国道三水--封开段上等级改造，畅通连接大西南的大动脉。基本完成西江大桥扩建主体工程。启动建设封开至郁南连接广梧高速公路跨江大桥。研究筹备三茂铁路的改线改造。加快通县通镇公路上等级和山区县连接周边地区的公路干道改造，扎实推进通行政村公路硬底化工程。加快建设肇庆新港一期工程和疏浚西江河道，努力创建西江南江口至肇庆段全国文明样板航道。积极争取肇庆火力发电厂一期６０万千瓦火力发电项目通过立项审批，抓紧建设大旺、白土、金利、广宁等一批２２万伏输变电工程，加快城市配网改造工程建设，抓紧实施第三期农村电网改造，缓解电力供应不足。抓好城乡水利减灾防灾工程建设，重点推进城市防洪工程、中小型水库除险加固工程和以景丰联围为重点的江河堤围加固达标工程。

　　（三）落实可持续发展战略，增强发展后劲

　　加快城市规划和建设。按照珠三角城市区域总体布局，以建设包括广州、佛山、肇庆在内的珠三角中心都市圈为目标，推进花园式、生态型、现代化大城市建设。修编中心城区总体规划，编制全市概念性规划，加强"山、湖、江、城"资源整合利用，优化城区功能布局。推进中心城区东扩南连，打造"一江两岸"百万人口大城市。强化经营城市意识，改革城市建设体制，积极引进外资、民资参与城市建设，加快端州城区城东新区开发，研究解决新建住宅区中小学布点少等问题。改善中心城区路网结构和交通路牌标志。加快城区道路特别是重点项目的建设改造和"亮化"、"绿化"工程建设。加强城市管理，坚决拆除违章建筑，制止乱摆乱卖，整治小区环境卫生。加强土地利用的宏观控制和政策引导，完善土地储备机制，扩大土地储备总量，继续实施易地开发补充耕地政策，清理违规用地，加大力度处置闲置土地，促进土地合理、高效和可持续利用。

　　加强环境保护和综合治理。深入开展国家环境保护模范城市的创建活动。强化环保责任制，实施"治污保洁工程"，确保空气、水源、噪声等环境质量达标。以西江、绥江流域和星湖、鼎湖为重点，全面开展江河湖库的水环境综合整治。推进城市环保设施建设市场化、产业化，加快建设城市生活污水处理厂和垃圾无害化处理场，完成城区污水处理厂二期工程以及高要、四会污水处理厂建设。加强建设项目环保验收，对工业园区实行"两个一"环境管理，实行污染物排放登记许可制度。注意保护农村环境，防止污染产业向农村转移。加强环保执法监督管理，加大污染防治，清理整顿不法排污企业。

　　搞好生态保护和建设。扎实开展创建全国环境优美乡镇和林业生态县活动，加强自然保护区和生态村镇建设。抓好造林绿化，建设速生丰产林基地和重点生态公益林工程，加快"两江三线"（西江、绥江、国道３２１、３２４线和省道四连线）水源涵养林改造和生态公益林建设，营造北岭山混交林基地，启动建设全省最大、品种最多的绿化苗木生产基地。依法保护和合理开发各种自然资源。

　　进一步做好人口与计划生育工作。全面落实人口与计划生育法律法规，强化计划生育工作责任制，确保达到各项目标要求。加强计生基层基础建设，严格流动人口计划生育管理，加快计划生育综合改革和信息化建设步伐，创新人口与计划生育综合治理制度，全面提高人口素质。

　　（四）落实科教兴市战略，提高经济增长的质量和效益

　　努力建设教育强市。优先发展教育，加快赶上先进地区水平。深化农村义务教育体制改革，逐步解决农村教育欠债问题。加快中小学校布局调整和山区、老区的改薄工作，努力改善办学条件，高水平、高质量普及九年义务教育。加大高中特别是示范性高中的建设力度，抓紧扩建改造肇庆中学新校和县（市）区重点中学，争取２００７年前建成省一级学校１７所、市一级学校９所。实施多元化投资的办学体制，积极鼓励社会力量办学。整合中等职业技术教育资源，加快市工贸学校和市技师学院建设。支持肇庆学院扩大办学规模和提高教学水平。力争把肇庆卫校升格为专科院校。重视教育信息化基础设施建设，发展远程教育和网络教育。支持各种类型的继续教育，逐步构建起终身教育体系，促进学习型社会的形成。

　　着力增强科技创新能力。完成市及各县（市）区科技发展中长期规划的编制工作。增加科技投入，建设一批科技创新平台。继续做好各类科技计划的申报和实施工作。强化企业技术创新和科技投入的主体地位，完善技术创新、知识创新和科技成果转化体系，重视知识产权保护。抓好科技示范镇和专业镇试点。加快市科技中心、农业创新中心和农村科技信息村村通工程建设。继续抓好科技园区和项目建设，扩大完善民营科技园，加强与巴西、爱尔兰等国外高新科技园区的合作。

　　积极实施人才强市战略。树立科学的人才观，加强人才培养，健全各类人才选拔任用、考核评价、激励监督机制。建立和落实人才引进优惠政策，积极引进科技、管理等我市急需的中高级人才。优化人才服务，加快发展人才市场，营造人才干事创业的良好环境。

　　（五）落实"十项民心工程"切实解决困难群众生产生活问题

　　认真实施"十项民心工程"。坚持以人为本，建立"十项民心工程"层级分项工作责任制。抓好农村住房特困户的危房改造，逐步解决城镇特困户的住房困难。加快农村改水工程建设，切实解决农村缺水地区饮水难问题。落实促进就业和再就业的政策措施，进一步完善劳动力市场，加快就业信息平台建设，加强下岗职工和失业人员再就业培训，扩大输出农村富余劳动力。完善再就业技能培训，建立公益性岗位调控机制，积极扶持就业特别是"４０５０"人员就业。深化农村税费改革，解决有关遗留问题，减轻农民负担。继续清理整顿涉农收费，坚决制止农村中小学乱收费、农民建房乱收费。加大教育扶贫工作力度，积极解决农村贫困家庭学生上学难问题。建立城乡统筹的就业、医疗、户籍和社会保障制度。做好水库移民安置工作。加强劳动监察，严格执行最低工资制度。切实保护外来工合法权益，重点解决建设领域拖欠工程款和农民工工资问题，建立健全及时支付农民工工资的机制，从源头上防止新的拖欠。力争农村合作医疗人口覆盖率达到４０％以上。完善抚恤补助自然增长机制，提高优抚保障水平，解决重点优抚对象生活难、住房难和医疗难问题。扎实开展济困助残活动。加强城市生活无着、流浪讨乞人员救助管理。做好救灾救济工作。加强自然灾害的监测预防。

　　进一步完善社会保障体系。狠抓社会保险扩面征收，依法扩大包括非公有制企业和灵活就业人员在内的覆盖面，努力提高社保基金的征缴率。完善社会保险市级统筹。适当提高养老金待遇。完善失业保险制度，做好下岗职工基本生活保障与失业保险并轨的后续工作。健全社会医疗保险制度和工伤保险制度。确保困难企业职工参保。强化社会保险基金监管，建立风险预警机制，确保基金安全和养老金按时足额发放。进一步完善城乡最低生活保障制度，对符合保障标准的城乡困难居民实行应保尽保。做好农村五保供养工作。

　　二、深化改革，进一步完善社会主义市场经济体制

　　深化国有企业改革。以市直国有企业为重点，积极引入外资、民资，采取一切适合我市国企实际的改革形式和操作方式，实施一步到位的产权改革，力争基本完成国企改革工作。坚持改革和发展并重，抓好骨干支柱企业的改革发展，努力盘活国有企业存量资产。拓宽改革成本筹集渠道，增加专项改革资金，依法处置企业债务。在企业改革中，坚持以人为本，维护职工合法权益，妥善安置企业职工，促进下岗职工再就业，处理好档案管理等问题，确保改革平稳推进。组建市国有资产管理委员会，加强国有资产监督管理。完善国有企业激励机制和分配制度改革。继续推进集体企业改革，发展多种形式的集体经济。

　　完善现代市场体系。大力发展产权、劳动力、土地等要素市场。完善土地有形市场。整顿建筑有形市场。规范旧城改造，完善补偿安置政策。推进垄断行业改革。继续整顿和规范市场经济秩序。坚决打击制售假冒伪劣商品，严厉惩处传销和变相传销。加快建立以企业和个人为重点的社会信用体系。加快发展行业协会、中介服务组织，逐步健全社会服务体系。

　　创新财政管理制度。推进公共财政管理框架建设，强化部门预算、国库集中支付、政府采购、收支两条线管理。逐步理顺市、区两级事权和财权关系，以及金渡、金陶等工业园区的税收分成问题。加强对政府资源及公共资源的统筹管理，逐步将收益纳入财政管理。优化支出结构，确保公共财政支出需要。建立财政抗风险应急预警机制和财政支出绩效评价体系。落实开发性金融贷款借用还的各项监管制度。支持税务部门严格依法治税，深化征管体制改革，加大稽查监督力度，确保地方财政收入稳步有效增长。

　　推进投融资体制改革。进一步规范政府投资行为，积极引导和鼓励社会投资。支持上市公司和骨干企业发行企业债券，选择有条件的企业通过重组和股份制改造上市融资。探讨组建中小企业担保机构的有效途径，努力解决中小企业担保难问题。扩大企业投资自主权，全面推行社会投资基本建设项目登记备案制度。推动银企双方拓宽合作领域，提高合作水平。支持金融部门创新经营管理体制。

　　继续改革行政管理体制。构建结构合理、配置科学、程序规范、监督有力的政府运行机制。认真贯彻实施《行政许可法》，规范政府行政行为。按照省的统一部署，６月底前基本完成部分政府部门机构设置和职能分工调整工作。按照"三并一减"的要求，加快县镇机构改革。进一步推动政资、政企、政事、政社分开，强化政府社会管理和公共服务职能。完善政府重大社会经济事项的科学化、民主化、规范化决策程序。大力发展电子政务，实现行政服务中心一站式网上并联审批，提高审批效率。加快建立健全各种突发事件应急机制，增强政府应对公共危机的能力，逐步构建四台（１１０、１１９、１２０、１２２）合一的快速反应处置平台。实行自然资源开发利用、公共事业项目、特许经营权项目公开招标竞投。深化城市基层管理体制改革。稳步推进事业单位机构和人事制度改革。落实安全生产责任制，加大安全生产执法力度，全面推行注册安全主任制度，探索建立安全生产长效机制，坚决遏制安全事故发生。

　　三、加快建设文化名市，促进政治文明、精神文明和社会各项事业全面发展

　　加强文化名市建设。做好文化名市规划和专家论证。深化文化体制改革，实现文化与经济、文化与旅游、文化产业与社会各项事业发展的互动互促。深入挖掘历史文化内涵和民间地方文化特色，规划建设一批文化项目，重点拓展宋文化和包公文化，打造特色文化精品。规划建设宋城景区，年内要打通宋城墙的朝天门，修建宋艺术品一条街。筹备举办首届肇庆端砚文化博览会。开拓文化市场，开发文化产品，拓展文化招商，加快发展文化产业。抓好文化基础设施建设，筹建市图书馆、博物馆，改造群众艺术馆。重视发展群众文化和民间艺术，支持山区、基层文化建设，丰富群众文化生活。加快建设广播电视光纤联网工程。加强文物保护。强化文化市场管理。

　　加强精神文明建设。深入学习贯彻"三个代表"重要思想和十六大精神，切实用"三个代表"重要思想武装头脑、指导实践、推动工作。大力创建全国文明城市。继续贯彻《公民道德建设实施纲要》，进一步加强和改进未成年人思想道德建设。开展"现代肇庆人精神"宣传和各种主题教育、道德实践活动。全面提高各类文明创建水平。继续推进"四进社区"活动。加强非公有制经济领域的文明创建工作。抓好千村生态文明工程建设。

　　加强社会各项事业建设。完善公共卫生体系，健全突发性公共卫生事件应急处理机制；加快市疾控中心和市卫监所移地重建工作，筹备建设市第一人民医院新院；深化药品集中采购招投标制度改革；大力改造农村薄弱乡镇卫生院，提高农村预防保健和基本医疗服务水平；加快中医药事业发展。培育发展体育产业和体育市场，提高竞技体育水平，广泛开展全民健身活动，完成"肇庆星湖山水体育健身环"的构建工作。发挥外事、侨务、台办对外交流的桥梁作用，进一步扩大与国际知名城市的友好往来。抓好全民国防教育，提高国防动员和民兵预备役工作水平，做好双拥共建和优抚安置工作。加大殡葬改革力度。积极推进行政区划调整工作，加强地名管理。完善防空设施建设管理。认真实施《肇庆市妇女发展规划》和《肇庆市儿童发展规划》。继续做好民族、宗教、统计、档案、气象、地方志、老龄、残疾人等工作。

　　加强社会治安综合治理。严密防范和坚决打击"法轮功"等邪教组织、各类刑事犯罪活动和经济犯罪活动，扫除黄赌毒，打击非法传销和地下六合彩活动。发挥群防群治作用，推进社会治安防控体系建设，建立健全治安防范长效机制，保障人民安居乐业。做好人民内部矛盾纠纷排查调处，积极预防和妥善处置群体性事件。

　　加强民主法制建设。自觉接受人大及其常委会的监督，认真落实人大及其常委会的决议、决定，及时向人大及其常委会报告工作。积极支持人民政协履行政治协商、民主监督、参政议政职能，认真听取民主党派、无党派民主人士和人民团体的意见。切实办好人大议案、人民代表建议和政协提案。建立和健全重大事项社会公示和听证制度，发挥经济技术顾问团作用，推进决策民主化、科学化、制度化。组织开展"四五"普法教育。加强政府法制工作，加大行政执法监督力度，提高依法行政水平。进一步提高政务公开的质量和水平。完善城市社区居民自治、村民自治和职工代表大会制度。认真做好人民来信来访工作。

　　加强政府作风和廉政建设。坚持"两个务必"，切实做到"五个力戒"和"干净干事"，进一步加强机关作风建设，强化公务员的学习培训，创建学习型机关，提高公务员的从政能力和服务水平。加强调查研究，深入体察民情。狠抓工作落实，建立完善岗位目标管理责任制和考核考评制度，抓好重点工作跟踪督办。创新机关作风评议方法，开展机关作风测评，督促后进单位切实改进作风。认真贯彻实施《中国共产党党内监督条例（试行）》和《中国共产党纪律处分条例》，切实抓好政府系统机关的廉政建设和反腐败斗争。落实廉政责任制，进一步规范事权、财权和人事权，加大行政监察和审计监督力度，强化监督约束，切实从源头上预防和治理腐败。坚决查处违法违纪案件，狠刹损害群众利益的不正之风。

　　各位代表！全市人民对我们充满着信任与期待。我们要在中共肇庆市委的正确领导下，振奋精神，求真务实，与时俱进，狠抓落实，推进改革，注重效益，为推动肇庆加快发展、跨越发展、协调发展积极工作，为实现我市全面小康社会和建设现代化新肇庆的目标而努力奋斗！
